# Improving Blueberry Anthocyanins’ Stability Using a Ferritin Nanocarrier

**DOI:** 10.3390/molecules28155844

**Published:** 2023-08-03

**Authors:** Wuyang Huang, Xingyu Zhao, Zhi Chai, Daniela D. Herrera-Balandrano, Bin Li, Yiyun Yang, Shan Lu, Zhigang Tu

**Affiliations:** 1 School of Food and Biological Engineering, Jiangsu University, Zhenjiang 212013, China; wuyanghuang@hotmail.com (W.H.);; 2 School of Life Sciences, Jiangsu University, Zhenjiang 212013, China; 3 Institute of Agro-Product Processing, Jiangsu Academy of Agricultural Sciences, Nanjing 210014, China; sophia_chai@163.com; 4 School of Life Sciences, Nantong University, Nantong 226007, China; 5 College of Food Science, Shenyang Agricultural University, Shenyang 110866, China; 6 Zhejiang Lanmei Technology Co., Ltd., Zhuji 311899, China; 7 The First Affiliated Hospital of Nanjing Medical University, Nanjing 210029, China

**Keywords:** anthocyanins, blueberry, stability, ferritins, nanocarrier, encapsulation

## Abstract

Blueberries are fruits known for their high level of anthocyanins, which have high nutritional value and several biological properties. However, the chemical instability of anthocyanins is one of the major limitations of their application. The stability of blueberry anthocyanin extracts (BAEs) encapsulated in a ferritin nanocarrier was investigated in this study for several influencing parameters, including pH, temperature, UV–visible light, redox agents, and various metal ions. The outcomes supported the positive role of protein nanoparticles in enhancing the stability of blueberry anthocyanins by demonstrating that the stability of encapsulated BAE nanoparticles with ferritin carriers was significantly higher than that of free BAEs and a mixture of BAEs and ferritin carriers. This study provides an alternative approach for enhancing blueberry anthocyanin stability using ferritin nanocarrier encapsulation.

## 1. Introduction

Blueberries are one of the most popular fruits [1], containing nutrients such as sugar, vitamins, mineral elements, and trace elements, in addition to high-value functional compounds such as anthocyanins [2]. Blueberries have several biological properties, such as preventing brain nerve aging, enhancing the body’s immune system, and protecting eyesight, which might be attributed to the anthocyanins [3,4]. Anthocyanins are the most effective natural water-soluble antioxidants found to date. They have been reported to activate the intracellular antioxidant defense system, protect cells from oxidative damage, and help prevent a variety of free-radical-related diseases, including alleviating metabolic syndrome [5].

However, in addition to the chemical structure of anthocyanins, numerous factors during processing and storage can affect the stability of anthocyanins and cause their degradation, which seriously affects their bioavailability, thereby limiting the large-scale use of blueberry anthocyanins [6]. Anthocyanins, as functional pigments, are susceptible to degrading, leading to the loss of color and biological activity [7,8]. Several environmental factors can significantly affect the stability of anthocyanins, including temperature, light, oxygen, enzymes, and pH, especially a neutral or alkaline pH [9,10,11]. Therefore, improving anthocyanins’ stability is foundational to ensuring that their functional properties are exploited, and has become a crucial issue that requires an urgent solution in the field of functional food research.

In the past few years, different methods have been proposed to improve the stability and bioavailability of anthocyanins, such as microencapsulation, protein complexes, and nanoparticle coating strategies [12,13,14]. Microencapsulation methods usually employ chitosan, carboxymethyl starch, xanthan gum, gum arabic, whey protein, and cyclodextrin capsules, providing a protective effect, improving the stability of anthocyanins, and ensuring their stability and sustained release [15,16,17]. The formation of protein complexes by adding α-casein, β-casein, β-lactoglobulin, whey protein, or soy protein in food matrices can be used as an effective way to delay and reduce the degradation of blueberry anthocyanins, thereby improving their biological stability under different conditions [18,19,20]. Nevertheless, nanoencapsulation processes have shown great potential in improving the effectiveness of delivery and the stability of biological compounds, including anthocyanins [20,21]. The anthocyanin-loaded nanocomposites prepared from chitosan hydrochloride, carboxymethyl chitosan, lactoglobulin, and lecithin cholesterol nanoliposomes exhibited greater stability at different storage temperatures and pH values than free anthocyanins [22,23,24]. In addition, nanocarrier systems for blueberry anthocyanin components can also be developed through co-encapsulation methods, which can more effectively incorporate anthocyanins [25].

Ferritin is an important iron-storage protein that plays a role in regulating the balance of iron metabolism in organisms; its interior stores a large amount of non-uniformly mineralized iron nuclei composed of iron hydroxide and phosphate [26]. The reduction and removal of the internal mineralized iron core results in a protein shell. The reversible dissociation assembly process of ferritin subunits regulated by environmental pH can be used to carry and embed anthocyanins into the ferritin nanocavity reactor [27,28]. Therefore, ferritin was used in this study to construct a protein nanocarrier to encapsulate blueberry anthocyanins, and the in vitro stability of blueberry anthocyanins encapsulated with protein nanocarrier was investigated and compared with the free anthocyanins.

## 2. Results and Discussion

### 2.1. Identification of Main Anthocyanin Composition of BAEs

In our previous study [29], the components of blueberry anthocyanins were qualitatively identified as delphinidin-3*-O-*galactoside, delphinidin-3*-O-*glucoside, cyanidin-3*-O-*galactoside, delphinidin-3*-O-*arabinoside, cyanidin-3*-O-*glucoside, petunidin-3*-O-*galactoside, petunidin-3*-O-*glucoside, peonidin-3*-O-*galactoside, petunidin-3*-O-*arabinose, peonidin-3*-O-*glucoside, malvidin-3*-O-*galactoside, malvidin-3*-O-*glucoside, and malvidin-3*-O-*arabinose (Figure S1), which showed the same elution order with similar content ratios. These 13 anthocyanins were detected in almost all blueberries of different cultivars, but in varying amounts [30].

### 2.2. The Stability of Blueberry Anthocyanins

#### 2.2.1. Effects of pH, Temperature, Light Type, and Redox Agents on the Stability of BAEs

**Figure 1:**
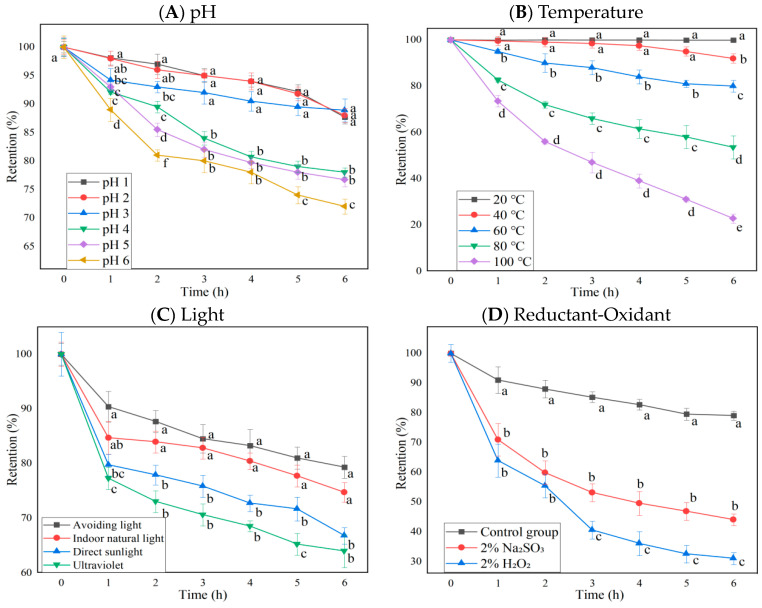
The influence of pH, temperature, light, and redox agents on the retention rate of blueberry anthocyanin extracts (BAEs) during six-hour storage. (**A**) pH: pH 1, 2, 3, 4, 5, and 6; (**B**) temperature: 20, 40, 60, 80, and 100 °C; (**C**) light: no light, indoor natural light, direct sunlight, ultraviolet light; (**D**) redox agent: 2% Na_2_SO_3_ and 2% H_2_O_2_. BAEs without an added redox agent were used as a control group. Data are represented as the mean ± standard deviation (SD, *n* = 3). Different lowercase letters indicate significant differences in BAE retention rate with different treatments at the same storage time (*p* < 0.05).

The anthocyanin retention rate changes of BAEs at different pH, temperature, light types, oxidant H_2_O_2_, and reductant Na_2_SO_3_ during six-hour storage are shown in Figure 1. The results indicated that pH value, temperature, light type, oxidant/reductant regent, and storage time were significant influencing factors on anthocyanin retention rates (*p* < 0.0001 for row and column factors), while their interaction was also significant (*p* < 0.0001). Blueberry anthocyanins were more stable in strongly acidic conditions (pH ≤ 3), since BAEs remained at 88.2 ± 1.2% retention rates after 6 h of storage. However, in the weakly acidic environment of pH 4–6, blueberry anthocyanins degrade much faster and more significantly, reaching 78.0 ± 0.8, 76.7 ± 1.2, and 72.0 ± 1.3% retention rates, respectively, after 6 h (Figure 1A). This is consistent with the findings reported by Lv et al. [31] and Zhang et al. [32], who studied the effect of pH value on the content of strawberry pigment and black bean skin anthocyanins, finding the stability of anthocyanins gradually decreased with the increase in pH values. Different pH conditions can change the molecular structure of anthocyanins, leading to color changes [12]. When pH < 2, anthocyanins exist as red to orange flavylium cations; from pH 3 to 6, anthocyanins exist as colorless chalcones or carbinal pesudobases; and when pH > 7, they change to purple to violet or blue quinonoidal bases. Due to different structural forms, the retention rate of anthocyanins varies.

Blueberry anthocyanins were more stable in low-temperature conditions (temperature ≤ 40 °C) since BAEs still maintained a retention rate of 97.5 ± 2.0% after 4 h of storage and only decreased by 8.0 ± 2.0% after 6 h. In a high-temperature environment, the degradation rate of blueberry anthocyanins was faster and the retention rates dropped, reaching 80.0 ± 2.5, 53.5 ± 5.0, and 22.7 ± 2.0% after 6 h at 60, 80, and 100 °C, respectively, with significant differences among them (*p* < 0.05, Figure 1B). Zhang et al. [33] found that the loss rate of mulberry anthocyanins reached 100% after treatment at 60 °C for 72 h. Changes in temperature over time resulted in anthocyanins converting to the chalcone structure, thereby decreasing the retention rate of anthocyanins [34]. Our results were consistent with the previous findings, regarding the effect of pH and temperature on strawberry and black bean skin anthocyanins [31,32]. Therefore, it is recommended that blueberry anthocyanins should be extracted, purified, processed, and preserved in an acidic and low-temperature environment.

Blueberry anthocyanins were most stable in dark conditions, with a retention rate of about 79.3 ± 2.0% after storage for 6 h, followed by indoor natural light (74.7 ± 1.8%). The anthocyanin retention rate under direct sunlight (66.7 ± 1.5%) and UV light (63.9 ± 3.0%) conditions was significantly lower than under no light or indoor natural light conditions (*p* < 0.05, Figure 1C). Du et al. [35] and Li et al. [36] also found anthocyanidins from *Vitis amurensis* and *Sorbus nigricans* had higher stability in the dark. This might be caused by light triggering a series of free radical reactions in singlet or triplet anthocyanins, leading to the degradation of anthocyanins [37]. Therefore, it is preferable to store blueberry anthocyanins in darkness to prevent their photodegradation.

The retention rates of blueberry anthocyanins in 2% H_2_O_2_ and 2% Na_2_SO_3_ solutions showed significant differences from the control (*p* < 0.05, Figure 1D). Under the action of H_2_O_2_, the BAE retention rate was only about 30% after 6 h. This might be because hydrogen peroxide can attack the C2 on the anthocyanin molecule and break its C3 bond, leading to the cleavage of the pyran ring, generating ester compounds and coumarin derivatives, destroying the structure of anthocyanins and reducing their stability [38]. Under the action of the reducing agent Na_2_SO_3_, the BAE retention rate was higher than that of the oxidant H_2_O_2_ (*p* < 0.05), but it also declined. After 6 h, the BAE retention rate was about 44.0 ± 2.0% (lower than the control, *p* < 0.05), which might be related to the bleaching property of SO_3_^2−^. Na_2_SO_3_ is prone to generating SO_2_, bleaching free anthocyanins [39]. The retention rate of anthocyanins decreased rapidly when different concentrations of hydrogen peroxide and sodium sulfite were added, which was consistent with a previous report by Li et al. [36].

Studies show that the stability of anthocyanidin boosted the anticancer activity of blueberry anthocyanidin in HeLa cells [40]. The retention time of blueberry anthocyanidin in vivo was extended following chitosan modification, and its antioxidant activity was enhanced, according to in vitro release and digestion simulations [41].

#### 2.2.2. Effects of Different Metal Ions and Concentrations on the Stability of BAEs

**Figure 2:**
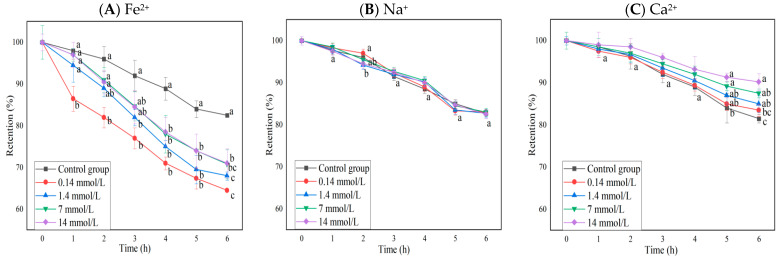
The influence of different ions and concentrations (0.14, 1.4, 7, 14 mmol/L) on the retention rate of blueberry anthocyanin extracts (BAEs) during six-hour storage. (**A**) Fe^2+^; (**B**) Na^+^; (**C**) Ca^2+^. BAEs without added ions were used as a control group. Data are represented as the mean ± standard deviation (SD, *n =* 3). Different lowercase letters indicate significant differences in BAE retention rate with different treatments at the same storage time (*p* < 0.05).

The changes in the anthocyanin retention rate of BAEs in different ion solutions at different concentrations during six-hour storage are shown in Figure 2. Fe^2+^ and Ca^2+^ had significant effects on anthocyanin retention rates (*p* < 0.0001), but Na^+^ did not (*p* > 0.05), and interactions between rows and columns were not significant (*p* > 0.05). The presence of Fe^2+^ decreased the retention rate of blueberry anthocyanins significantly (*p* < 0.05), and the retention rate of BAEs treated with a low concentration of FeSO_4_ decreased more rapidly than that treated with a high concentration. Furthermore, with the increase in Fe^2+^ concentration, the decrease in BAE retention rate became slow and weak but still greater than that of the control group (*p* < 0.05), whereas the presence of Ca^2+^ appeared to be beneficial for maintaining the stability of BAEs. Only high concentrations of CaCl_2_ (≥7 mmol/L) could significantly increase the retention rate of blueberry anthocyanins during long storage periods (>5 h). Guo et al. [42] also reported that Fe^2+^ had a significant fading effect on purple cabbage anthocyanins, while Zhang et al. [43] and Li et al. [44] found that Ca^2+^ had the effect of protecting and stabilizing anthocyanin extracts during the extraction process, which was consistent with our results for blueberry anthocyanins.

### 2.3. The Improvement of BAE Stability using Ferritin Nanocarriers

#### 2.3.1. Examination of Encapsulated BAE Nanoparticles with Ferritin Carriers

**Figure 3:**
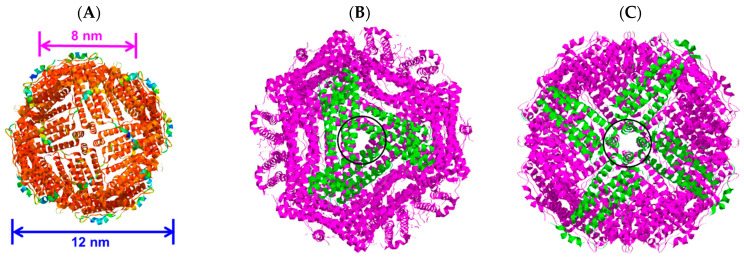
The three-dimensional structure of ferritin molecules: (**A**) ferritin shell; (**B**) triple axis channel; and (**C**) quadruple axis channel.

**Figure 4:**
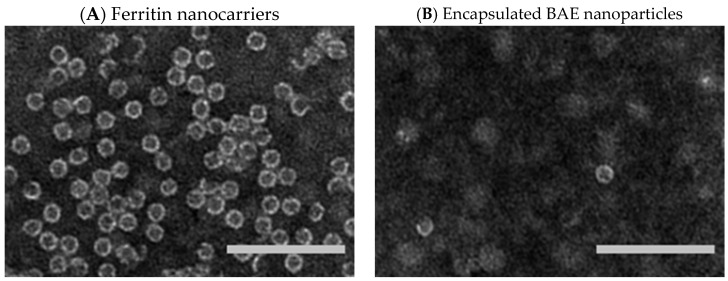
Transmission electron microscope images. (**A**) ferritin nanocarriers; and (**B**) encapsulated blueberry anthocyanin extract (BAE) nanoparticles. Scale bar at 100 nm.

The protein ferritin is present in humans, plants, and other organisms. It oversees iron storage and elimination. Iron homeostasis can be maintained in a soluble, non-toxic, bioavailable form [45,46]. The ferritin shell is a highly symmetric dodecahedral hollow spherical molecule composed of 24 subunits with a particle size of 12 nm and an 8 nm cavity (Figure 3A, ferritin structure was drawn by RasMol 2.6. The X-ray crystallographic structure of the ferritin was obtained from the Protein Data Bank (http://www.rcsb.org/pdb, accessed on 27 July 2023)), 8 triple axis channels (Figure 3B), and 6 quadruple axis channels (Figure 3C), which provides a structural basis for the carrier to perform its embedding and delivery functions. Some nanocarriers are difficult to obtain, but ferritin nanocarriers can be produced in *Escherichia coli*, which has cost advantages and high feasibility and can be used to obtain heat-resistant purified products [47]. To date, ferritin has been used to encapsulate anthocyanins to maintain their stability [48]. In this study, ferritin nanocarriers were successfully obtained by removing ferrous ions. A transmission electron microscope (TEM) image showed that ferritin carriers were mostly spherical, with a hollow structure in the center of the protein shell and a protein carrier particle diameter of about 15 nm (Figure 4A), which was a nanocarrier. The ferritin shell had some channels formed by ferritin subunits and subunits spiraling along the rotation axis in the dodecahedral structure of the ferritin shell nanocavity. These channels connect the internal cavity of ferritin with the external environment, with a pore diameter of about 0.5 nm, which is necessary for metal ions, oxygen, and small molecules to enter and exit the ferritin cavity [49,50]. After embedding BAEs, the encapsulated BAE nanoparticles were observed using a TEM. It could be seen from the image that the central cavity of the protein carrier disappeared and a high number of anthocyanins were embedded in the central cavity (Figure 4B).

#### 2.3.2. Effects of Ferritin Nanocarriers on the Stability of BAEs

**Figure 5:**
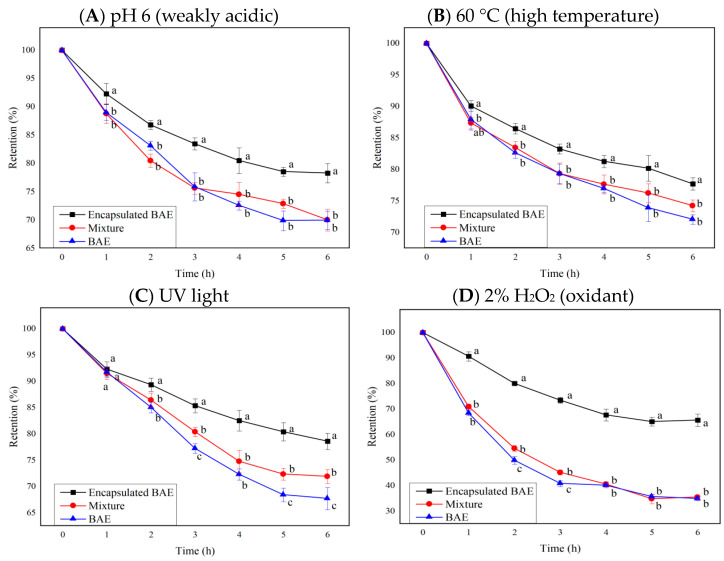
The influence of ferritin nanocarriers (encapsulated BAE nanoparticles and the unencapsulated mixture) on the retention rate of blueberry anthocyanin extracts (BAEs) during six-hour storage. (**A**) pH 6; (**B**) 60 °C; (**C**) ultraviolet (UV) light; (**D**) 2% H_2_O_2_. Data are represented as the mean ± standard deviation (SD, *n =* 3). Different lowercase letters indicate significant differences in BAE retention rate with different treatments at the same storage time (*p* < 0.05).

Tests on the stability of blueberry anthocyanins revealed that numerous exogenous factors, such as pH value, temperature, light radiation, ionic strength, oxidants, and reducing agents, affect the stability of anthocyanins and cause their degradation. Based on the most influential indicators in stability analysis, the stability of encapsulated BAE nanoparticles was tested and compared with that of free BAE and the unencapsulated mixture of BAEs and ferritin carriers at pH 6, 60 °C, and 2% hydrogen peroxide, and under ultraviolet light. The change curves of the retention rate of free anthocyanins, a simple mixture of carrier and anthocyanin molecules, and encapsulated BAE nanoparticles are shown in Figure 5, where results showed *p* < 0.0001 for interaction, row, and column factors, except interaction *p* > 0.05 in Figure 5B.

The anthocyanin retention rate of encapsulated BAE nanoparticles was significantly greater than that of free BAEs and a simple mixture of BAEs with ferritin carriers (*p* < 0.05 vs. mixture and BAEs) during six-hour storage in weakly acidic, high temperature, UV light, and oxidant regent conditions. However, there was no significant difference between the mixture and BAEs under most conditions (*p* > 0.05). Mixing with ferritin carriers improved the stability of BAEs under UV light for some time (*p* < 0.05 vs. BAEs at 3, 5, 6 h). After 6 h of storage, encapsulation with ferritin nanocarriers could inhibit 27.7 ± 6.4, 20.2 ± 3.4, 33.7 ± 4.1, and 47.2 ± 1.1% degradation of BAEs under the conditions of pH 6, 60 °C, UV light, and 2% H_2_O_2_, respectively. Therefore, the encapsulated BAE nanoparticles possessed better stability, protecting anthocyanins from degradation under weakly acidic pH, high temperature, light radiation, and oxidant conditions. Previous reports also found that embedding resveratrol in ferritin nanoparticles could improve their photothermal stability and antioxidant activity [51]. The degradation rate of astaxanthin could be reduced from about 40% to 3% by preparing ferritin-astaxanthin-embedded materials, demonstrating the feasibility of using nano-ferritin to improve the stability of small molecule compounds [52]. In this study, the stability of blueberry anthocyanins was successfully improved by embedding BAEs into ferritin nanocarriers, improving the pH stability, heat resistance, light resistance, and antioxidant reduction properties. Ferritin nanocarriers had a certain protective effect on anthocyanins, which might be attributable to the protein shell of ferritin isolating the interference of adverse external environmental factors on the internal anthocyanin molecules [48]. Simultaneously, it was speculated that the amino acid residues in the cavity of ferritins could also interact with anthocyanin molecules, stabilizing and protecting them [24].

## 3. Materials and Methods

### 3.1. Materials, Chemicals, and Reagents

The blueberry anthocyanins were extracted from Rabbiteye blueberries (Lishui Town, Nanjing, China) [53] and stored at −18 °C at the Institute of Agricultural Products Processing, Chinese Academy of Agricultural Sciences. High-performance liquid chromatography (HPLC) solvents potassium chloride and acetonitrile were acquired from Sinopharm Chemical Reagent Co., Ltd. (Shanghai, China) and TEDIA (Fairfield, OH, USA), respectively. Ferritin (from horse spleen), MOPS buffer, 2,2-bipyridine, and bovine serum protein (BSA) were procured from Sigma-Aldrich (Shanghai, China). The bicinchoninic acid (BCA) and protein-assay kits were purchased from Beijing Solarbio Company (Beijing, China). The reagents used in the experiment are all of analytical grade.

### 3.2. HPLC Analysis

The composition of blueberry anthocyanin extracts (BAEs) was analyzed according to our previous method using HPLC analysis performed on an Agilent-1200 (Agilent Technologies, Santa Clara, CA, USA) with an XDB-C18 column (250 mm × 4.6 mm, 5μm) [29]. Mobile phase A was 1% phosphoric acid buffer, while mobile phase B was acetonitrile. Gradient elution was conducted with a 20 μL injection volume and a 0.6 mL/min flow rate. After 90 min of elution, anthocyanins were detected at 520 nm and identified by comparing them with previously reported literature [30].

### 3.3. Preparation of Encapsulated BAEs with the Protein Nanocarrier

The protein nanocarrier was obtained from horse ferritin. Under anaerobic conditions, a ferritin carrier (1 mM) with sodium bisulfite (3% *w*/*v*) in 50 mM Mops buffer (pH 7.0) was prepared; therefore, iron ions were reduced to ferrous ions, and ferrous ions were removed using dialysis. Furthermore, 1 mM 2,2-bipyridine was used to precipitate the residual ferrous ions and was centrifuged at 5000 rpm to remove the precipitate. The obtained supernatant was dialyzed three times with a buffer solution to remove excess 2,2-bipyridine. With BSA as the standard, the concentration of the target protein nanocarrier was calculated using an ultraviolet spectrophotometer and the Lowry method [54]. The carrier solution obtained was kept at 4 °C before use. The mixed BAE was obtained by mixing it directly with the carrier solution.

**Figure 6:**
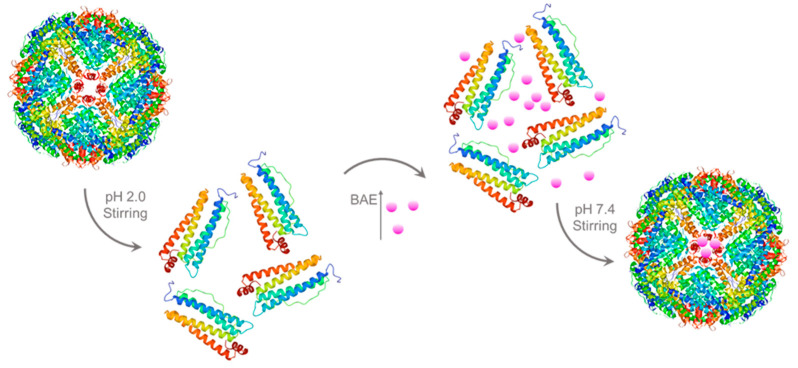
Schematic illustration of preparation process of encapsulated blueberry anthocyanin extracts (BAEs) with the ferritin nanocarrier.

For encapsulated BAEs, the ferritin solution (2 μM) was adjusted to pH 2.0 by slowly adding 1.0 M hydrochloric acid and then incubating at 4 °C with magnetic stirring for 20 min to dissociate ferritin into free subunits. Afterward, 10 mM BAE was slowly added to a 2 μM protein carrier solution in a volume ratio of 1:11 and placed in a beaker packed with tinfoil in darkness. The pH was adjusted to neutral with 1.0 M NaOH, and the solution was stirred in the dark for 2 h. After stirring, the solution was placed into PBS buffer (pH 7.4) for dialysis for 24 h to remove free BAE (molecular weight cutoff (MWCO) = 12,000–14,000 Da). After that, BAE was encapsulated in a ferritin carrier to obtain the nanoparticle (2 μM). The preparation process of encapsulated BAEs is shown in Figure 6 (The X-ray crystallographic structure of ferritin was obtained from the Protein Data Bank (http://www.rcsb.org/pdb, accessed on 27 July 2023). The ferritin structure was drawn by RasMol 2.6.

### 3.4. Transmission Electron Microscope Observation

The morphology of the protein nanocarrier and BAE nanoparticle was observed using an HT7700 Hitachi Hi-Tech Transmission Electron Microscope (Japan Hi-Tech Company, Tokyo, Japan). Briefly, an aliquot was taken and dropped onto the surface of a carbon Formvar-coated copper grid. After drying at room temperature, samples were stained negatively with 2% aqueous phosphotungstic acid for 30 s. Then, the samples were observed under a transmission electron microscope with an accelerating voltage of 80 kV. The images were presented under 60,000× magnification.

### 3.5. Determination of the Total Anthocyanin Content and Retention

The total anthocyanin content of free, mixed, or encapsulated BAEs during the stability test was determined by the pH differential method [55]. Before determination, the encapsulated BAE sample’s pH was adjusted to 2.0 to release the anthocyanins. Potassium chloride buffer (pH 1.0) and sodium acetate buffer (pH 4.5) were used to dilute the samples. The absorbance of each dilution was measured at 520 nm and 700 nm using a UV-6300 visible spectrophotometer (Shanghai Media Instrument Co., Ltd., Shanghai, China). The total anthocyanin content was calculated according to the following formula. The anthocyanin retention rate (%) was the percentage of total anthocyanin content after storage divided by the total anthocyanin content before storage, and is expressed as milligram per gram of dry weight basis (mg/g DW).

(1) $$The\;total\;anthocyanin\;content\;(mg/g\;DW)=\frac{\left(A\times MW\times DF\times V\times 1000\right)}{\left(\varepsilon \times L\right)\times Wt}$$

where A is value of absorbance, A = (A_520nm_ − A_700nm_) pH 1.0 − (A_520nm_ − A_700nm_) pH 4.5, MW is the molecular weight of cyanidin-3-glucoside (449.2 g/mol), DF is the dilution ratio, V is solvent volume (L), 1000 is the factor for conversion from g to mg, ε is the molar extinction coefficient (26,900 L/mol cm), L is the cuvette length (cm), and Wt is the dry weight of the sample (g).

### 3.6. Stability Test

The stability test was carried out on various factors, including pH, storage temperature, light conditions, oxidants, reductant agents, and salt ion type concentration. The pH stability of the free BAE was evaluated by adjusting the pH to 1, 2, 3, 4, 5, and 6, respectively, with HCl and NaOH and determining the total anthocyanin content and retention rate every hour during the storage for a total of 6 h. The stability of free BAEs was investigated for various storage temperatures (20, 40, 60, 80, and 100 °C), light conditions (no light, indoor natural light, direct sunlight, and ultraviolet (UV) light), 2% H_2_O_2_, 2% Na_2_SO_3_, and different salt ions at a range of concentrations (NaCl, CaCl_2_, and FeSO_4,_ each at 0.14, 1.4, 7, and 14 mmol/L). The light conditions for these mentioned experiments were as follows: for UV light, the samples were placed 30 cm under UV light from a 40 W fluorescent lamp (Beijing Research Institute of Electric Light Source, Beijing, China) and the UV intensity was 0.375 μW/m^2^, detected by a UV radiation meter (Photoelectric Instrument Factory of Beijing Normal University, Beijing, China); no light meant in the dark; and indoor natural light meant the samples were placed under natural light without any fluorescent lamps. Based on the most influential indicators of stability analysis, the BAE nanoparticle was evaluated and compared to the stability of pH 6, high temperature (60 °C and 80 °C), 2% H_2_O_2_, and UV light with the free BAE and unencapsulated BAE mixture. The figures of BAE retention rate change during the stability test (Figure 1, Figure 2 and Figure 5) were obtained by using OriginLab OriginPro v8.5 SR1 (Northampton, MA, USA).

### 3.7. Statistical Analysis

All data were expressed as the mean ± standard deviation (SD) of three independent experiments. The statistical analysis was carried out using GraphPad Prism Version 8 (GraphPad Software, Inc., La Jolla, CA, USA). Ordinary one-way analysis of variance (ANOVA) was performed to compare the different treatment groups using Tukey’s multiple comparisons tests. An ordinary two-way ANOVA was performed to analyze the statistical significance of the row factor (storage time), column factor (different treatment), and their interaction. When the *p*-value was less than 0.05, it was considered to be statistically significant. Different lowercase letters indicate significant differences in BAE retention rate with different treatments at the same storage time (*p* < 0.05).

## 4. Conclusions

In this study, a ferritin nanocarrier with an approximate particle diameter of 15 nm was obtained. In addition, BAE was encapsulated with a ferritin nanocarrier, and the stability of encapsulated and free anthocyanins was compared. The results demonstrated that free BAEs are more stable at low pH (pH 3) and low temperatures (40 °C), as well as in the dark. On the other hand, encapsulated BAEs protected anthocyanins from degradation under weakly acidic pH (pH 6), high temperature (60 °C), ultraviolet light, and oxidant conditions. The results revealed that the stability of encapsulated BAE nanoparticles with ferritin carriers was considerably greater than that of free BAEs, demonstrating the advantageous role of protein nanoparticles in enhancing the stability of blueberry anthocyanins. The protein nanocarrier developed in this study would have the potential to be utilized for the delivery of BAEs as functional constituents with enhanced chemical stability; however, further efficiency experiments should be performed and limitations of the high cost should be considered and settled in the future.

## Data Availability

The data presented in this study are available upon request.

## Supplementary Materials

The following supporting information can be downloaded at: https://www.mdpi.com/article/10.3390/molecules28155844/s1, Figure S1: HPLC chromatogram of rabbiteye blueberry anthocyanin extracts and their identification.
