# Supplementary material for: Improving Blueberry Anthocyanins’ Stability Using a Ferritin Nanocarrier
Source: Molecules. 2023 Aug 3;28(15):5844. doi: 10.3390/molecules28155844 (PMC10421234; doi:10.3390/molecules28155844)
Supplement: Supplementary file 1 [file molecules-28-05844-s001.zip › molecules-2501977-supplementary.pdf]

Figure S1. HPLC chromatogram of rabbiteye blueberry anthocyanin extracts and their identification.

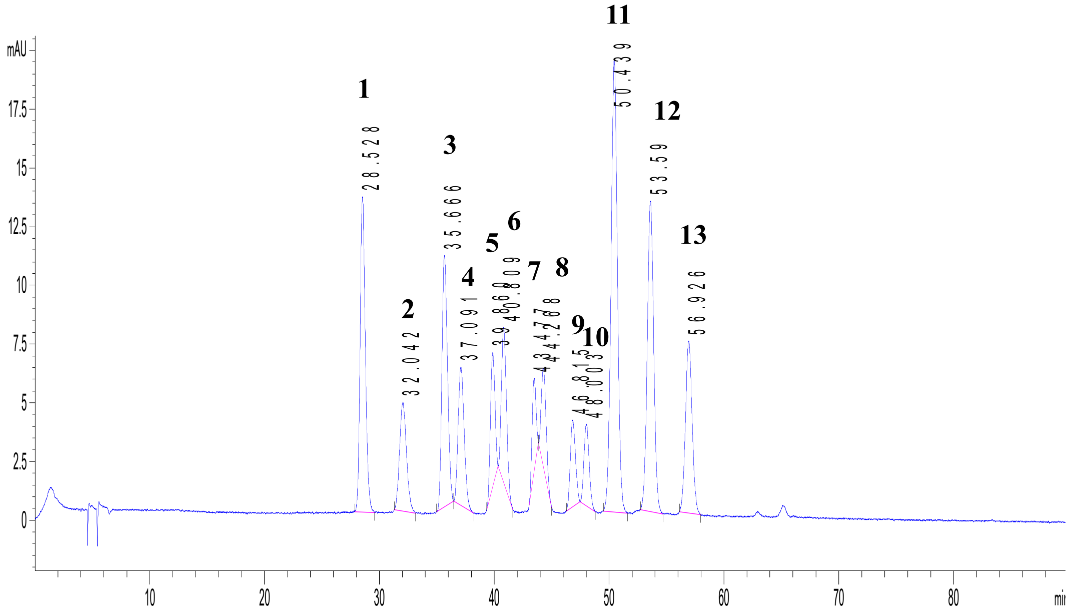

Peak	Retention time (min)	Identified anthocyanins
1	28.528	Delphinidin-3-*O*-galactoside
2	32.042	Delphinidin-3-*O*-glucoside
3	35.666	Cyanidin-3-*O*-galactoside
4	37.091	Delphinidin-3-*O*-arabinoside
5	39.86	Cyanidin-3-*O*-glucoside
6	40.809	Petunidin-3-*O*-galactoside
7	43.477	Petunidin-3-*O*-glucoside
8	44.268	Peonidin-3-*O*-galactoside
9	46.815	Petunidin-3-*O*-arabinose
10	48.003	Peonidin-3-*O*-glucoside
11	50.439	Malvidin-3-*O*-galactoside
12	53.598	Malvidin-3-*O*-glucoside
13	56.926	Malvidin-3-*O*-arabinose
